# STROBE-compliant article: Is continuity of care associated with avoidable hospitalization among older asthmatic patients?

**DOI:** 10.1097/MD.0000000000004948

**Published:** 2016-09-23

**Authors:** Yu-Hsiang Kao, Shiao-Chi Wu

**Affiliations:** Institute of Health and Welfare Policy, National Yang-Ming University, Taipei, Taiwan.

**Keywords:** asthma, avoidable hospitalization, continuity of care, geriatrics

## Abstract

Continuity of care (COC) has a proven relationship with health care outcomes. However, evidence regarding an association between COC and avoidable hospitalization for elderly patients with asthma is insufficient.

A retrospective cohort study was performed using Taiwanese National Health Insurance claim data from 2004 to 2013. Patients were retrospectively followed for 2 years; the COC index (COCI) for asthma was measured in the 1st year, and avoidable hospitalization for asthma and follow-up time were determined in the subsequent year. Cox proportional hazards regression was employed to examine hazard ratios (HRs) between COC and avoidable hospitalization for asthma after adjustment for confounding factors. Adjusted HR (aHR) was also calculated by stratifying each variable to investigate whether the effect of COC on hospitalization for asthma was avoidable and how this varied across levels of COCI.

Of 3356 patients included in this study, 1648 patients (49%) had a COCI of 1, and the average COCI was 0.73. Compared with patients with high COC (COCI = 1), those with low COC (COCI < 0.5) had a significantly higher risk of avoidable hospitalization for asthma (aHR = 2.68; 95% confidence interval [CI]: 1.55–4.63). In addition, after stratified analysis, we determined that COC plays a much more important role for patients who were women, had low insurance premiums, and had no comorbidities.

High continuity of ambulatory asthma care is linked to a reduced risk of avoidable hospitalization for asthma in elderly asthmatic patients.

## 1 Introduction

Asthma is a common chronic respiratory disease,^[1]^ and poor asthma control affects patient's quality of life and is a frequent cause of asthma-related hospitalization.^[2]^ In addition, asthmatic patients spent much more medical expenditures than patients without asthma.^[3]^ Continuity of care (COC) is a core element of primary care,^[4,5]^ and it represents an ongoing therapeutic relationship between a patient and care provider that is characterized by trust and responsibility.^[6,7]^ The stronger the ongoing physician–patient relationship is during the treatment of chronic diseases, the higher is the likelihood of reducing risks of unnecessary hospitalization,^[8–12]^ emergency department (ED) visits,^[13–16]^ and health care costs.^[17]^ Because of the disease characteristics of asthma, previous studies have often highlighted the effect of COC on asthmatic children or adolescents.^[14,15,18]^ The findings of these studies have shown that implementing higher levels of COC would result in a lowered prevalence of hospitalization for asthma. Investigating whether these findings could be applied to older patients with asthma is warranted. To our knowledge, only 1 Korean study has been conducted involving COC in asthmatic elderly patients, and this study reported that elderly asthmatic patients aged 65 to 84 years had lower risks of hospitalization, ED visits, and care expenses if the associated COC was improved.^[19]^

Ambulatory care-sensitive conditions (ACSCs) are conditions for which satisfactory outpatient care can potentially reduce the risk of subsequent hospitalization.^[20,21]^ Therefore, such conditions are also referred to as “preventable hospitalization” or “avoidable hospitalization.”^[22,23]^ Also, this concept corresponds to quality, accessibility, and performance of ambulatory care in the healthcare system.^[21,24]^ Although several previous studies have examined the relationship between COC and avoidable hospitalization,^[9,10,12,25]^ there is currently no empirical evidence on the relationship between COC and avoidable hospitalization for asthma among older asthmatic patients. The aim of this study is to investigate the relationship between COC and the risk of subsequent hospitalization for elderly patients with asthma by using the population-based database of the Taiwanese National Health Insurance (NHI) system.

## 2 Methods

### 2.1 Study sources

Taiwan's NHI program was launched in March 1995. It provides universal, compulsory, and nationally administered health insurance that enhances the public's accessibility to health care services, and the program currently includes over 99% of the Taiwanese population.

To identify the study population, a retrospective cohort study was conducted using the Longitudinal Health Insurance Database 2010 (LHID2010) maintained by the National Health Research Institute in Taiwan. The database consists of 25 sets of 40,000 people randomly selected from the entire population of NHI enrollees; in 2010, the total sample comprised approximately 1 million beneficiaries. No significant differences exist in the distribution of beneficiaries’ basic characteristics, such as age and sex, between this dataset and the entire population in 2010.^[26]^ The database stores unique encrypted identification for each patient, in addition to the patient's sex, date of birth, medical professional consulted, and International Classification of Diseases, Ninth Revision, Clinical Modification (ICD-9-CM) codes for each medical encounter. Data accuracy and information pertaining to patient diagnosis, as retrieved from the database, were previously validated.^[27]^ The protocol for this study was approved by the Institutional Review Board of the National Yang-Ming University of Taiwan (IRB Approval Number: YM103047E).

### 2.2 Study population

In this study, patients aged 65 years or older diagnosed with asthma (ICD-9-CM codes 493.xx) between January 1, 2005 and December 31, 2011 were identified from the database. Patients associated with at least 2 ambulatory visits, or at least 1 asthma-related hospital admission, were eligible for inclusion.

The earliest date of diagnosis was defined as the index date. Exclusion criteria for the patients are outlined as follows: withdrawal from the NHI program during the study period (n = 326); having experienced inpatient asthma care prior to, or during the COC period (n = 39), because the process of continuity of ambulatory care could be impacted by the experience of hospitalization^[10,28]^; or less than 4 outpatient visits during the COC period (n = 4452), because a small number of visits caused unstable COC index (COCI) estimates.^[9,28,29]^

**Figure 1:**
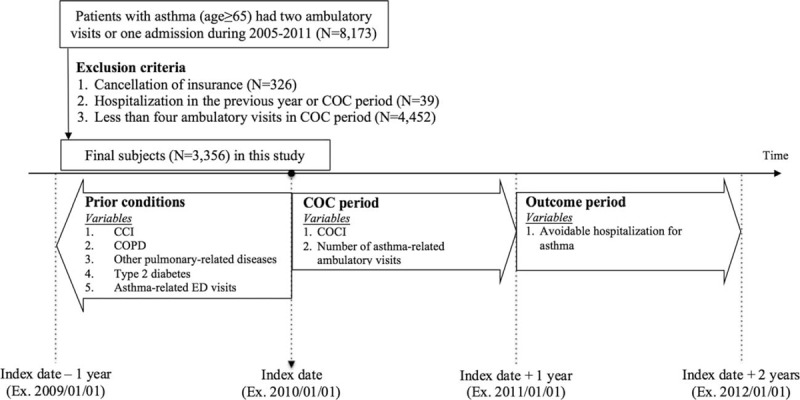
A framework and flow chart for the study. CCI = Charlson comorbidity index, COC = continuity of care, COCI = continuity of care index, COPD = chronic obstructive pulmonary disease, ED = emergency department.

A total of 3356 patients were finally recruited in this study. To avoid time-dependent bias and incorrect conclusions obtained by simultaneously measuring COC and health outcomes,^[30]^ all patients (n = 3356) were followed for 2 years after the index date. The first year was denoted as the COC period and the subsequent year the outcome period (Fig. 1).

### 2.3 Variable definitions

#### 2.3.1 Dependent variable

The Agency for Healthcare Research and Quality provided a definition for prevention quality indicators,^[31]^ identifying hospital admission for asthma using a code pertaining to the main diagnosis (ICD-9-CM code, 493.xx). Therefore, in this study, avoidable hospitalization was defined as an event that occurred during the outcome period. The follow-up time was defined as the number of days from the date of the end of the COC period to the occurrence of the avoidable hospitalization for asthma. However, if no avoidable hospitalization for asthma occurred, the patient was censored at the end of the outcome period.

#### 2.3.2 Independent variable

The COCI score, used as the independent variable, was measured during the COC period. The score ranges from 0 to 1 (values close to 1 represent a greater COC) and measures the dispersion of contact between patient and physician.^[32]^ The COCI has been widely adopted in studies based on health care claim databases,^[4,9,14,15,19,25]^ because it is less sensitive to the number of physician visits and is suitable for application to a large amount of outpatient visit data.^[25]^

The general formula is 
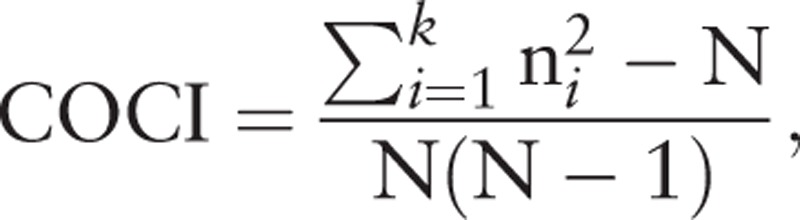


where N is the total number of physician visits, n_*i*_ is the number of visits to the *i*_th_ physician, and *k* is the total number of physicians. In this study, the total number of physician visits (N) and the number of visits to a given physician (n_*i*_) included ambulatory claims for asthma as the major diagnosis. The patients were categorized into three groups based on the first and third quartile value of COCI as follows: low, medium, and high.^[25,29]^

#### 2.3.3 Confounding factors

Confounding factors were identified in 3 mutually exclusive periods. First, variables measured on the index date were sex,^[9,33,34]^ age,^[9,33,34]^ and insurance premium (<20,000 NTD, 20,000–40,000 NTD, and ≥40,000 NTD), which was used as a proxy indicator of income status.^[35]^ Second, the variables measured in the year prior to the index date included chronic obstructive pulmonary disease (COPD) (ICD-9-CM codes 491, 492, or 496),^[36]^ pulmonary-related diseases (ICD-9-CM codes 490, 494, or 495),^[36]^ diabetes mellitus (DM) (ICD-9-CM code 250),^[36]^ the Charlson comorbidity index (CCI), and the number of asthma-related ED visits. The CCI and number of asthma-related ED visits were used as proxy indicators of health status^[9]^ and disease severity.^[25]^ The CCI score contains 17 categories of comorbid conditions defined by ICD-9-CM codes, and it is calculated according to enhanced ICD-9-CM coding algorithms.^[37]^ Third, because patients’ health status during the COC period also may impact the outcome, the number of asthma-related ambulatory visits was used as a proxy for patients’ health status.^[9,19]^

#### 2.3.4 Statistical analysis

In this study, descriptive statistical analysis was used to present the distribution of patient characteristics. In addition, chi-squared tests and one-way analysis of variance were used to analyze associations between patient characteristics and COC.

The Cox regression model assumes that the ratio of the hazards of two subjects is the same at all times; in this study, the scaled Schoenfeld residual was used to test whether this assumption was valid.^[38]^ With the valid proportional hazard assumption (*P* = 0.7921), the Cox regression model was applied to examine the association between COC levels and the risk of avoidable hospitalization for asthma among elderly patients. Multivariate analysis was used to calculate adjusted hazard ratios (aHRs) by adjusting for sex, age, insurance premium, COPD, pulmonary-related diseases, DM, CCI, number of asthma-related ED visits, and number of asthma-related ambulatory visits. The variance inflation factor (VIF) is used to detect the presence of multicollinearity; a value greater than 10 indicates the severity of multicollinearity in the regression model. In our model, no multicollinearity was represented by a VIF of less than 5 in each variable. The aHR stratified by each variable was then calculated to investigate the effect of COC levels on the extent to which avoidable hospitalization for asthma.

Two-sided criteria with *P* values of less than 0.05 were considered to be statistically significant in this study. All statistical analyses and data management were conducted using SAS software version 9.4 (SAS Institute, Cary, NC).

## 3 Results

The average COCI of patients was 0.73 ± 0.30 (data not shown in the table). Of 3356 patients, 1648 (49.0%) were in the high COC group (COCI = 1), 851 (25.4%) in the medium COC group (COCI = 0.5–0.99), and 857 (25.7%) in the low COC group (COCI < 0.5). The characteristics of all patients and of each COC group are shown in Table 1. Variables were significantly related to the level of COC in age, history of COPD, and number of ambulatory visits (*P* < 0.05). Regarding avoidable hospitalization use during the outcome period, the percentage of patients who had avoidable hospitalization for asthma was 1.3% in the high COC group, 2.4% in the medium group, and 4.0% in the low COC group; therefore, the differences were statistically significant (*P* < 0.001).

**Table 1:**
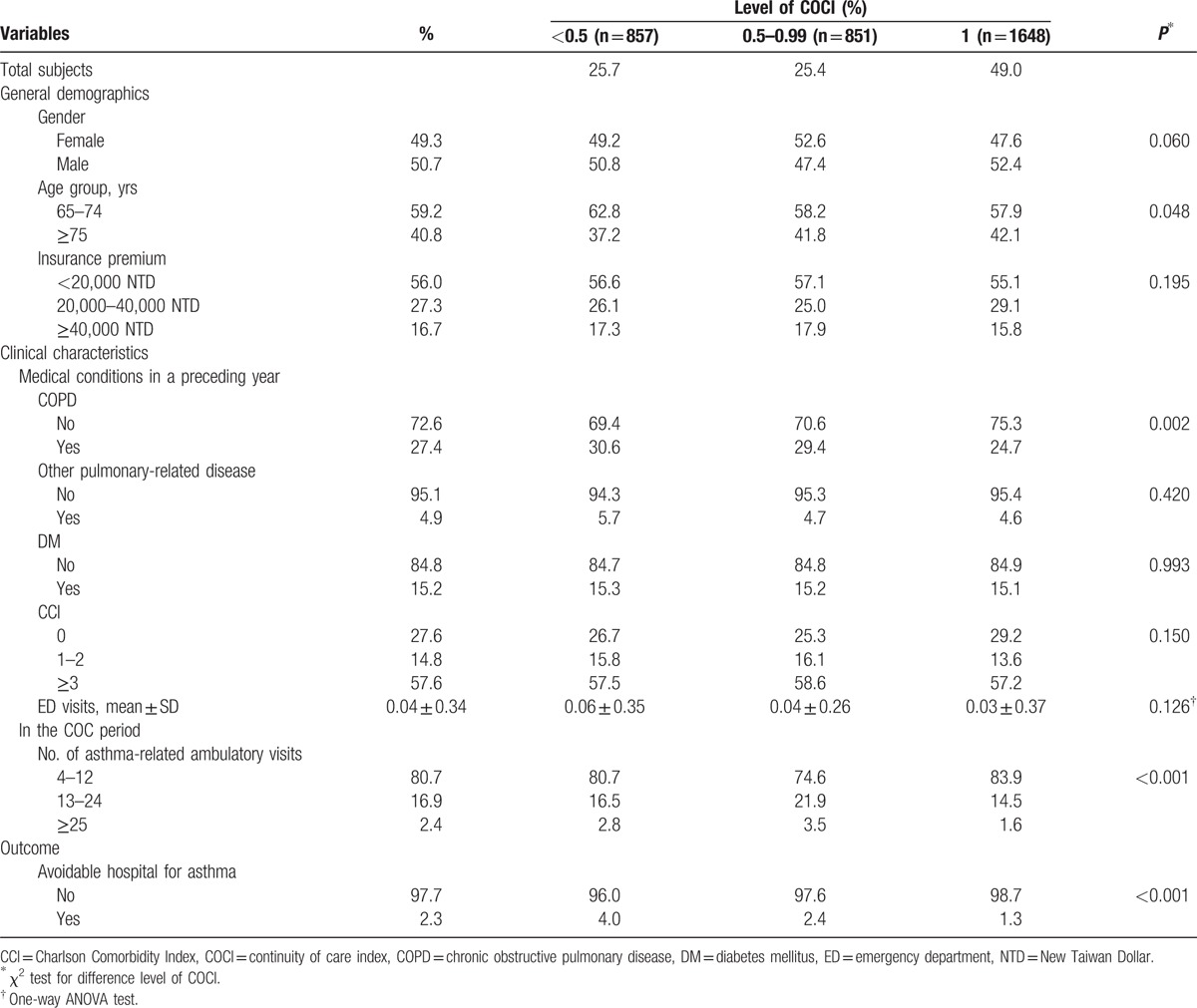
Characteristics in subjects (N = 3356) by continuity of care index group.

The results of Cox proportional regression are presented in Table 2. As a crude result, the risk of avoidable hospitalization because of asthma for patients in the low COC group was significantly higher than that for those in the high COC group (HR = 3.01; 95% CI, 1.76–5.15). After adjustment for other confounding factors, the adjusted risk for patients in the low COC group was significantly higher than that for those in the high COC group (aHR = 2.68; 95% CI, 1.55–4.63). Despite there being no statistical significance for the medium group compared with the high COC group, results showed an increased risk tendency of avoidable hospitalization for asthma for patients in the medium group, whether the crude or adjusted models were used (aHR = 1.77, 95% CI, 0.96–3.24; aHR = 1.49, 95% CI, 0.80–2.75, respectively). We observed no significant interaction between the COC groups and other variables in the model.

**Table 2:**
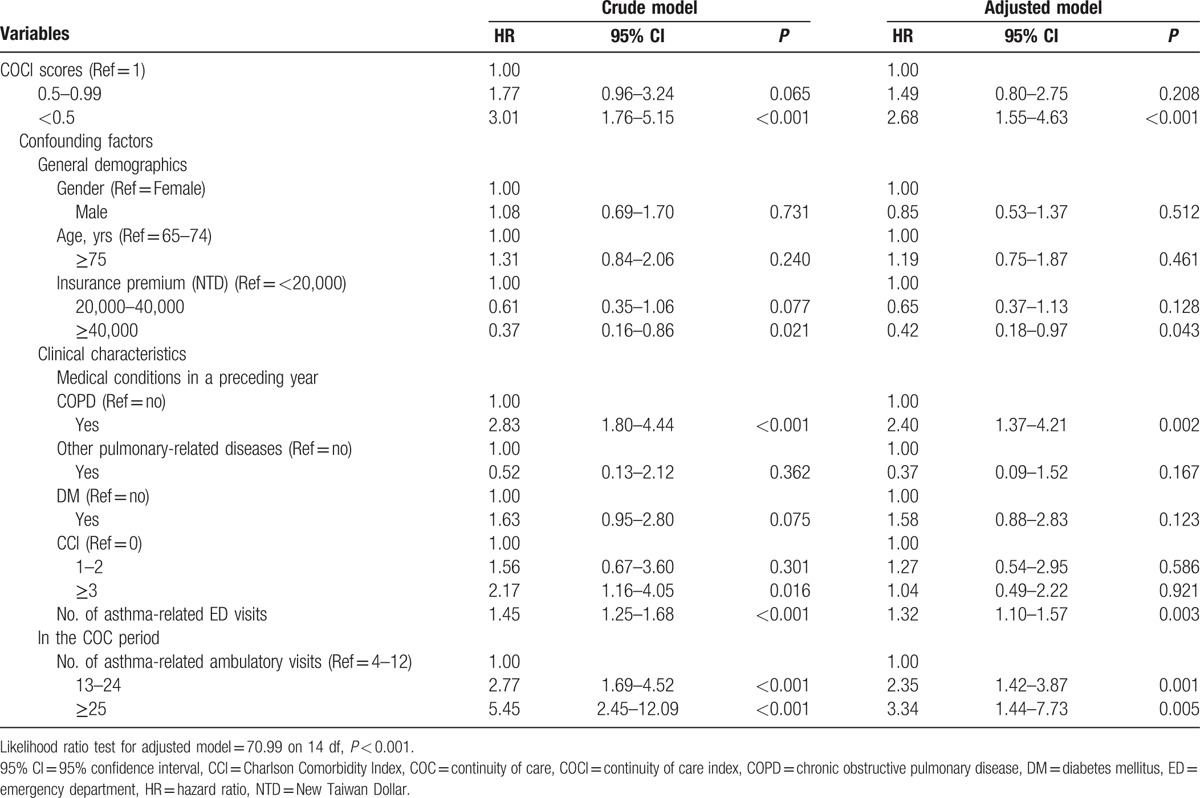
Factors associated with avoidable hospitalization for asthma among elderly patients (N = 3356).

Table 3 shows the risk for avoidable hospitalization due to asthma in elderly asthmatic patients stratified by each variable. After analyzing each variable by controlling the other variables, we determined that patients with low COC had a statistically significant higher risk of avoidable hospitalization for asthma compared with those who had high COC scores. This included the following groups: women patients (aHR = 3.48, *P* < 0.01), patients aged 65 to 74 years (aHR = 3.10, *P* < 0.01), patients aged ≥75 years (aHR = 2.54, *P* < 0.05), patients with an insurance premium <20,000 NTD (aHR = 2.48, *P* < 0.01), patients with an insurance premium 20,000 to 40,000 NTD (aHR = 4.14, *P* < 0.05), patients with no COPD history (aHR = 4.18, *P* < 0.001), patients without any other pulmonary-related diseases (aHR = 2.92, *P* < 0.001), patients with no DM (aHR = 2.74, *P* < 0.01), patients with a CCI score of zero (aHR = 10.09, *P* < 0.01), and patients with 4 to 12 ambulatory visits (aHR = 3.09, *P* < 0.01).

**Table 3:**
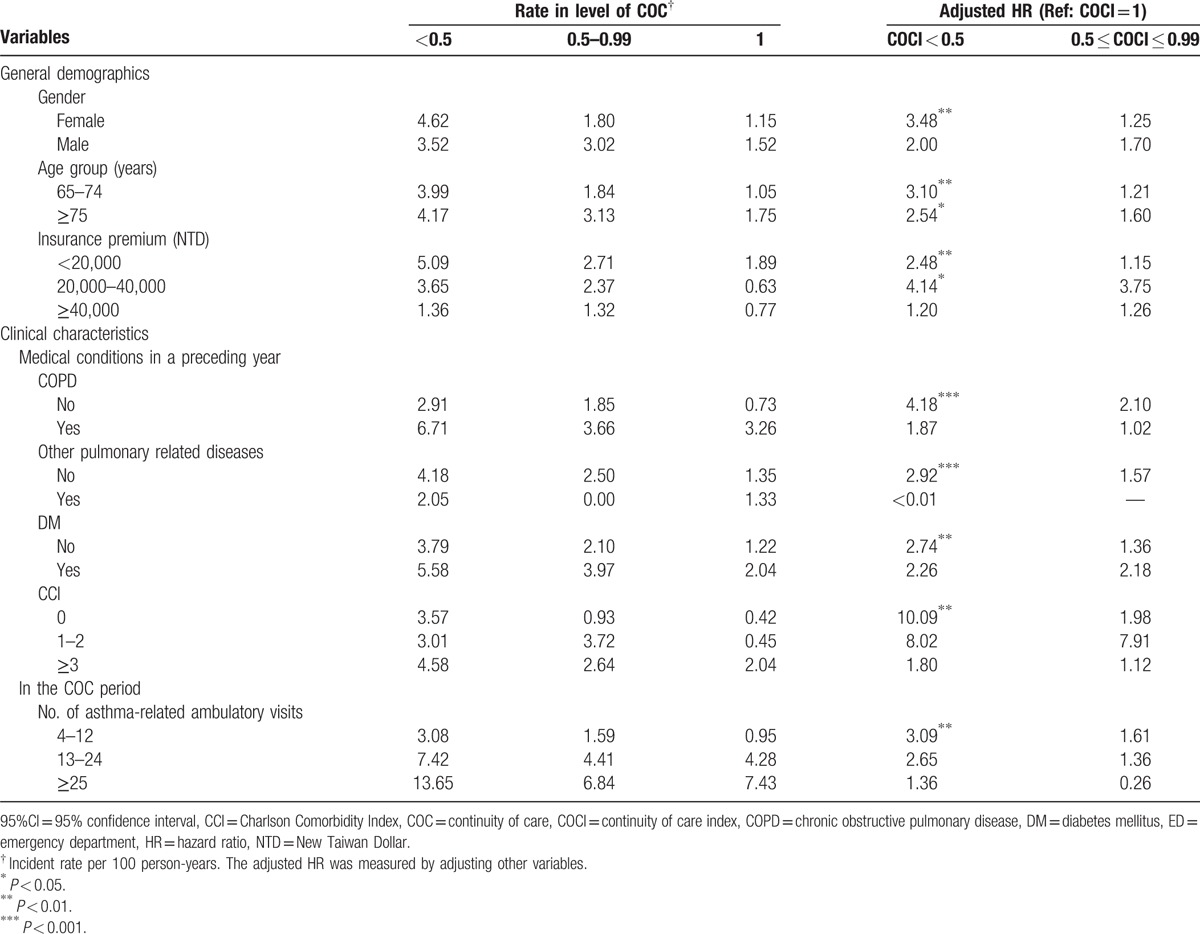
Risk of avoidable hospitalization for asthma between various COC groups by stratifying each variable (N = 3356).

## 4 Discussion

The data suggest that elderly asthmatic patients in Taiwan have a high continuity of ambulatory asthma care. The average COCI was 0.73 for elderly asthmatic patients, and half of patients received ambulatory asthma care from a single physician in the first observational year. The high COC score in Taiwan could be attributed to several factors. The Taiwanese government implemented an asthma pay-for-performance (P4P) program in 2001, with the aim of encouraging health care agencies to provide improved disease management for patients with asthma. Previous studies have demonstrated that asthmatic children in Taiwan^[15]^ have higher COC scores (average COCI = 0.68) than do those in the United States (average COCI = 0.39)^[14]^ and Canada (average COCI = 0.26).^[18]^

Asthma is the most common respiratory disease among children^[39]^ and aging adults.^[40]^ The continuity of treating asthmatic children with inhaled medicine provides safe and effective long-term disease management for asthma.^[41,42]^ Previous studies have suggested that superior COC was associated with lower hospitalization and ED use among children with asthma.^[14,15,18]^ Although the effect of COC may vary between different populations,^[5]^ our findings support that the effect of COC is also effective in reducing avoidable hospitalization for asthma for elderly populations.

Our findings support those of Hong et al^[19]^ who investigated the effect of COC in older patients in South Korea and identified an inverse association between COC and the risk of hospitalization for asthma for older patients. Their study calculated the COCI by using medical institution units and focused on 4 different chronic diseases (hypertension, COPD, asthma, and diabetes) among elderly patients. Their findings suggested that improved COC is associated with fewer hospital admissions for patients with asthma, and that it is more effective than in other diseases.

Many previous studies calculating COC for all diseases^[9,10,33,43]^ have reported that patients with superior COC had a lower risk of avoidable hospitalization. However, the effect of COC on health outcomes could be confounded when a study includes several diseases.^[44]^ Therefore, recent studies have focused on a single disease to clarify the association between COC and health outcomes. For example, Huang et al^[15]^ focused on an asthmatic children population and identified a significantly positive effect of COC in reducing asthma-related ED use. Hussey et al^[45]^ focused on Medicare beneficiaries with congestive heart failure, COPD, or diabetes and demonstrated that superior COC was significantly associated with lower odds of hospitalization for each of the 3 chronic conditions. Lin et al^[25]^ indicated that a high COC was associated with a lowered risk of COPD-related avoidable hospitalization among adults with COPD. Our study focused on elderly patients with asthma also found a significantly inverse relationship between COC levels and the risk of avoidable hospitalization for asthma. Therefore, we suggest that older asthmatic patients with low COC should endeavor to develop an ongoing relationship with a single physician to reduce the risk of avoidable hospitalization for asthma.

In terms of a stratified analysis of each variable, the data suggest that COC plays a much more important role for older asthmatic patients who were women, had low insurance premiums, and had no comorbidities. In studies focused on elderly people, the rates of avoidable hospitalization for women were found to be higher,^[46]^ and socioeconomic status (SES) was found to be adversely associated with avoidable hospitalization.^[47]^ Furthermore, the possibility of older asthma patients receiving care from other physicians was low because patients with no other comorbidities might only contact a physician when suffering an asthma attack. As a result, these patients may benefit from improving their own COC.

Our study demonstrates that COC plays a major role in the reduction of avoidable hospitalization for asthma among older asthmatic patients. The findings also support the fact that improving COC is favorable for both patients and the health care system.^[29]^ Therefore, we suggest that governments consider designing financial incentives for patients and physicians to increase motivation, thereby improving COC. A randomized clinical study proposed that offering financial incentives for both health care providers and patients could generate superior care outcomes for patients.^[48]^

This study has some limitations. First, a previous study reported that asthma severity is associated with a higher risk of hospitalization^[49]^; however, claimed data in the study did not include results of clinical pulmonary function tests such as spirometry, lung volume, and diffusing capacity to define disease severity and health status.^[50,51]^ In this study, we used asthma-related ED visits as a proxy for asthma severity^[25]^ and used CCI and frequency of ambulatory visits for asthma as proxy indicators of patients’ health status.^[9,19]^ Second, we could not collect data regarding patient educational level or household income, which may also affect the care continuity and outcome measurements.^[34,50]^ Nevertheless, we adopted information concerning patient insurance premiums as an SES indicator, as obtained from the claim database.^[52]^ Finally, our findings are related only to patients with more than 3 asthma-related outpatient visits per year.^[14,17]^

This study has several advantages. First, we found that the asthma P4P program might improve patients’ COCI under a universal insurance system. Second, COCI affects not only the health care outcomes of children or adolescents with proven asthma,^[14,15,18]^ but also the health outcomes of elderly asthmatic patients. Third, we measured COCI at a physician level, which may provide superior information about the association between COC and avoidable hospitalization among elderly asthmatic patients than that obtained from measurements at the level of health care institutions.^[43]^ Fourth, we focused on a specific disease and used critical criteria to identify study subjects, which is more sensitive in identifying the relationship between COC and health outcomes,^[29,44]^ thereby precisely determining the association between COC and avoidable hospitalization for asthma. Fifth, more than 99% of Taiwanese are enrolled in the compulsory NHI program; thus, the findings are highly representative of the whole population. Moreover, the nationwide administrative databases provide all clinical practices and decrease the effect of recall bias, thereby delivering superior results to those from national surveys,^[53]^ hospital-based data sets,^[54]^ or small area data sets.^[18]^ Finally, this study applied a longitudinal study design to follow all patients for 2 years and measured the condition of care continuity prior to the health care outcome. In addition, current study not only avoids the problem of cross-sectional design,^[10,33]^ but also proposes stronger evidence of the association between COC and avoidable hospitalization for asthma.

## 5 Conclusion

Our study shows that higher continuity of ambulatory asthma care for elderly asthmatic patients could reduce the risk of avoidable hospitalization for asthma. From a policy-making perspective, we recommend that policy makers create effective policies for older patients with asthma to strengthen the ongoing physician–patient relationship and improve disease-controlling ability.
